# Comparison of individual and pooled stool samples for the assessment of intensity of *Schistosoma mansoni* and soil-transmitted helminth infections using the Kato-Katz technique

**DOI:** 10.1186/s13071-015-1101-1

**Published:** 2015-09-24

**Authors:** Ashenafi Kure, Zeleke Mekonnen, Daniel Dana, Mitiku Bajiro, Mio Ayana, Jozef Vercruysse, Bruno Levecke

**Affiliations:** Public Health Laboratory, Southern Nations Nationalities and People’s Regional State Health Bureau, Hawassa, Ethiopia; Department of Medical Laboratory Sciences and Pathology, Jimma University, Jimma, Ethiopia; Department of Virology, Parasitology and Immunology, Ghent University, Merelbeke, Belgium

**Keywords:** Soil-transmitted helminths, *Schistosoma mansoni*, Infection intensity, Pooled samples, Mass drug administration, Cost-effectiveness

## Abstract

**Background:**

Our group has recently provided a proof-of-principle for the examination of pooled stool samples using McMaster technique as a strategy for the rapid assessment of intensity of soil-transmitted helminth infections (STH, *Ascaris lumbricoides*, *Trichuris trichiura* and hookworm). In the present study we evaluated this pooling strategy for the assessment of intensity of both STH and *Schistosoma mansoni* infections using the Kato-Katz technique.

**Methods:**

A cross-sectional survey was conducted in 360 children aged 5–18 years from six schools in Jimma Zone (southwest Ethiopia). We performed faecal egg counts (FECs) in both individual and pooled samples (pools sizes of 5, 10 and 20) to estimate the number of eggs per gram of stool (EPG) using the Kato-Katz technique. We also assessed the time to screen both individual and pooled samples.

**Results:**

Except for hookworms, there was a significant correlation (correlation coefficient = 0.53–0.95) between the mean of individual FECs and the FECs of pooled samples for *A. lumbricoides*, *T. trichiura* and *S. mansoni*, regardless of the pool size. Mean FEC were 2,596 EPG, 125 EPG, 47 EPG, and 41 EPG for *A. lumbricoides*, *T. trichiura*, *S. mansoni* and hookworm, respectively. There was no significant difference in FECs between the examination of individual and pooled stool samples, except for hookworms. For this STH, pools of 10 resulted in a significant underestimation of infection intensity. The total time to obtain individual FECs was 65 h 5 min. For pooled FECs, this was 19 h 12 min for pools of 5, 14 h 39 min for pools of 10 and 12 h 42 min for pools of 20.

**Conclusions:**

The results indicate that pooling of stool sample holds also promise as a rapid assessment of infections intensity for STH and *S. mansoni* using the Kato-Katz technique. In this setting, the time in the laboratory was reduced by 70 % when pools of 5 instead of individual stool samples were screened.

## Background

Soil-transmitted helminthiasis and schistosomiasis remain an important public health problem in several parts of the world [1]. The main strategy to control the morbidity caused by these helminthiasis is mass drug administration (MDA) programmes in which a single oral dose of benzimidazole drugs (soil-transmitted helminthiasis) and praziquantel (schistosomiasis) are periodically administered to school-aged children [2]. These programmes have recently received increased political and scientific attention [3, 4], with which the World Health Organization (WHO) aims to increase the coverage of the children in need of anthelminthic drugs from the current 34 % for soil-transmitted helminthiasis [5] and 13 % for schistosomiasis [6] to at least 75 % by 2020, and to ultimately eliminate both diseases as a public health problem in children [7].

This worldwide upscale of MDA programmes, however, creates the need for a monitoring system that allows programme managers, healthcare decision makers and donors of the drugs to assess whether objectives are being met, and if necessary, to adjust the strategy implemented [2]. Thus, it will be imperative to periodically re-assess helminth infections to determine whether the MDA programme progresses as anticipated. Moreover, these MDA programmes traditionally operate in resource-constrained settings, and hence it is indispensable that programme managers and healthcare decision-makers have some flexibility to minimize both financial and technical resources, while assuring a reliable assessment of the progress made.

A diagnostic strategy that could translate into substantial cost savings is pooling of samples. Our group has recently shown that examination of pooled stool samples using McMaster egg counting method provided soil-transmitted helminthiasis intensity estimates comparable to those obtained by individually examining stool samples [8]. However, it remains unclear whether this strategy also applies to other neglected tropical diseases and diagnostic techniques, and whether a pooled examination strategy is altogether reducing costs.

In the present study we compared the intensity of soil-transmitted helminths (STHs; *Ascaris lumbricoides*, *Trichuris trichiura* and hookworm) and *Schistosoma mansoni* infections obtained by both an individual and a pooled examination strategy. To screen the stool samples we used the Kato-Katz technique, which is the WHO recommended technique to quantify parasite eggs in stool [9, 10]. Finally, we assessed the time to screen both individual and pooled samples.

## Methods

### Ethical approval

The study was approved by the Research Ethics Review Board of Jimma University, Jimma, Ethiopia (refno RPGC/364/2014). The school authorities, teachers, parents, and children were informed about the purpose and procedures of the study. The written consent form was prepared in two commonly used local languages (Afaan Oromo and Amharic) and handed over to the children’s parents/guardians. Only those children who were willing to participate and whose parents or guardians signed the written informed consent form were included in the study. Moreover, an additional written informed consent form for children older than 12 years was obtained. A single-oral dose of albendazole (400 mg) and praziquantel (40 mg/kg) was administered to children excreting eggs of STH and *S. mansoni*, respectively [2].

### Study area and study population

The study was conducted in Jimma Town and Kore Village. Jimma town is the capital of Jimma Zone and is located at a latitude and longitude of 7^°^ 40′N36^°^ 50′E, 352 km southwest of Addis Ababa. It is characterized by a semi-arid type climate with an average annual rainfall of 800–2,500 mm. The mean daily temperature is 19 °C, and ranges from 12 to 30 °C.

Kore village is found in Mana district of Jimma Zone and located 32 km to the west of Jimma Town. The district is located at an average altitude of about 1,450 m above sea level. The district is generally characterized by warm climate with a mean annual maximum temperature of 25 °C and a mean annual minimum temperature of 18 °C. The annual rainfall ranges from 1,138 to 1,690 mm. Kore Health Center is the only health service providing facility (Report document 2013/2014 of Jimma zone administration). Our study focused on school children from age 5 to age 18. There were 11 and 4 schools in Jimma Town and Kore Village, respectively, in which all grades are represented.

### Study design

Between February and May 2014, a school based cross sectional study was carried out in 6 primary schools in both Jimma Town (3 schools) and Kore village (3 schools). These schools were purposively selected based on their prevalence of *S. mansoni* and STH infections [8, Bajiro *et al.*, unpublished data; Kore Health Center] and their proximity to water sources.

**Fig. 1:**
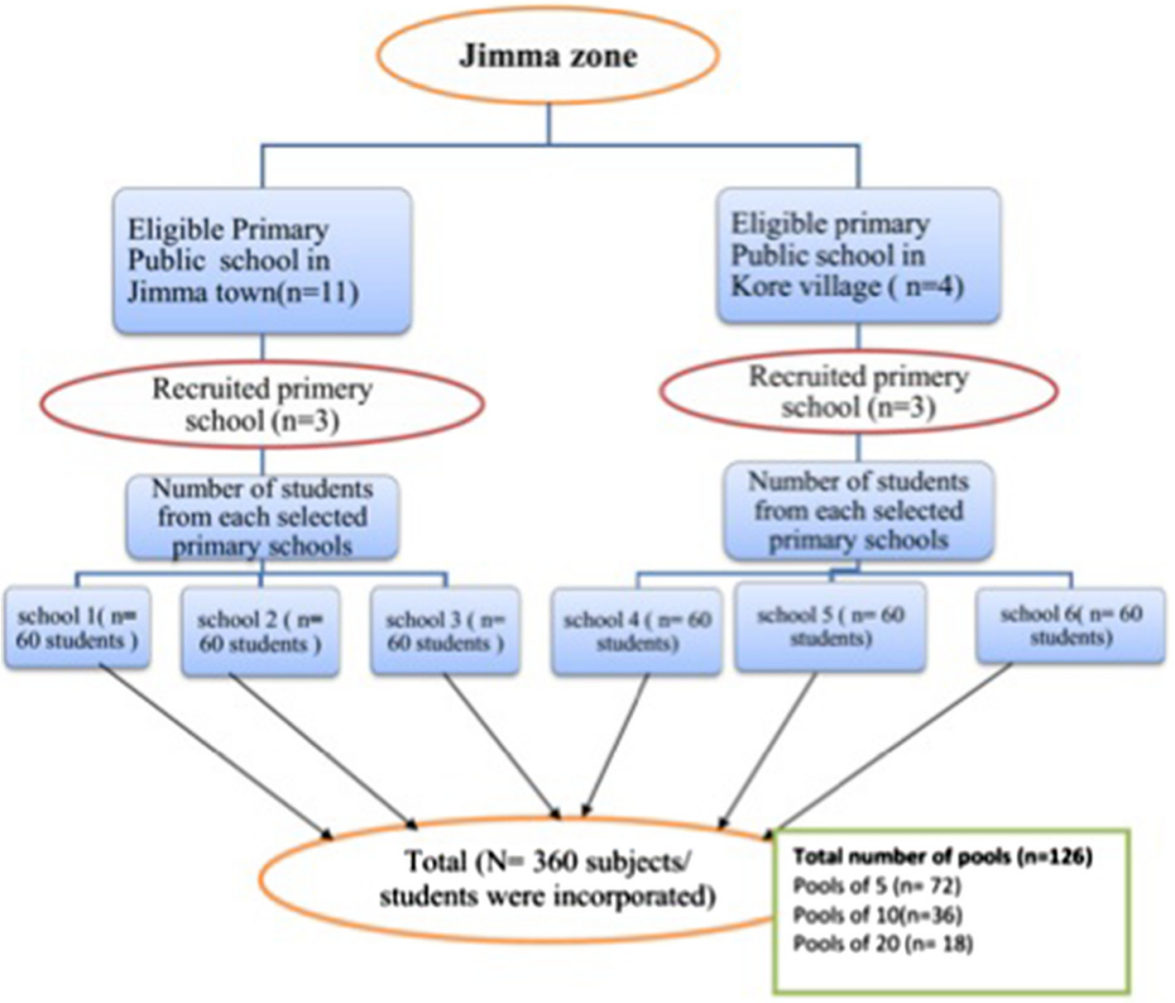
Number of schools and stool samples for assessing infection intensity of soil-transmitted helminths and *Schistosoma mansoni*

In each school, we stratified subjects according to three age groups (age 5–9 years, age 10–14 years and age 15–18 years). For each age group at least 20 subjects were randomly selected, resulting in a total of at least 60 subjects per school. The subjects were asked to provide at least 3 g of stool to examine the samples individually and to pool individual stool samples. All stool samples were individually processed by the Kato-Katz technique. Figure 1 illustrates the number of primary schools eligible, recruited, and included in the statistical analysis.

**Fig. 2:**
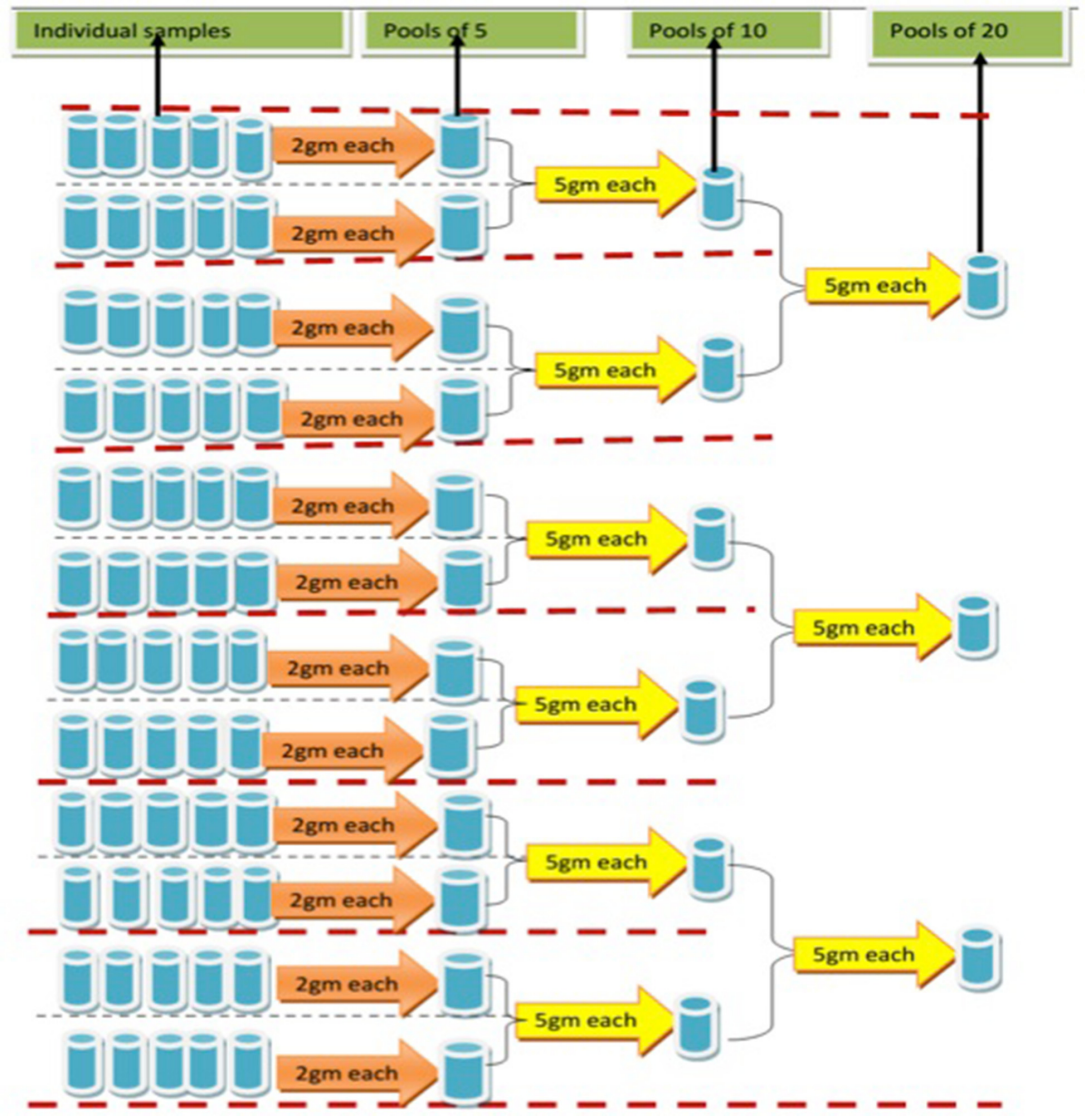
Procedure to obtain pools of 5, 10 and 20 individual stool samples. Sixty individual samples were arranged in 12 rows with each row consisting of 5 individual samples, subsequently 12 pools of 5, 6 pools of 10 and 3 pools of 20 individual samples, resulting in total of 21 pooled samples per school

In addition, stool samples were pooled in pools of 5, 10, and 20 individual samples. The general procedure for pooling individual samples is illustrated in Fig. 2, and is discussed in more detail below.

At first, the 60 individual samples collected from one school were randomly organized in 12 rows of 5 individual stool samples. For each row, 2 g of each of the 5 individual stool samples was transferred into a new pre-labelled plastic beaker (resulting in a total of 12 pools of 5 individual stool samples). After homogenization, 5 g from 2 plastic beakers representing pools of 5 individual samples were transferred into another new pre-labelled plastic beaker, resulting in a total of 6 pools of 10 individual samples. Next, 5 g was transferred from the 2 vials of pools of 10 into a new pre-labelled plastic beaker, resulting in 3 pools of 20 individual stool samples. Homogenization was standardized by means of stirring the stool until homogenized. Stools from different subjects have different colours. We stopped stirring the pooled stool when the pool had one homogeneous colour. A general tutorial on pooling of stool can be found at http://www.youtube.com/watch?v=IUZijtBABn0. Finally, each of the pools was processed by the Kato-Katz technique as done for individual samples.

### Parasitological examination

The Kato-Katz technique was applied to process all individual and pooled stool samples. Due to its simple format and ease of use in the field, the Kato-Katz technique is the diagnostic technique recommended by the WHO for the quantification of both STHs and *S. mansoni* eggs in stool [9–11]. A tutorial on how stool samples were examined using Kato-Katz can be found on https://www.youtube.com/watch?v=WpcZejHa_jM. A subset of 10 % of the smears were re-examined by a senior scientist to ensure quality of the parasitological examination.

### Assessment of time required to prepare and screen individual and pooled samples

We measured the time to prepare and screen both individual and pooled samples. To this end, we timed the time (i) to prepare Kato-Katz thick smears in batches of 10 (*n* = 36 + 12 = 48), (ii) to make one pool of 5 (*n* = 72), (ii) to make one pool of 10 from 2 pools of 5 (*n* = 36), (iii) to make one pool of 20 out of 2 pools of 10 (*n* = 18), and (iv) to count helminth eggs in a Kato-Katz thick smear (*n* = 360 + 126 = 486).

### Statistical analysis

#### Assessment of infection intensity

The infection intensity was determined for *A. lumbricoides*, *T. trichiura*, hookworms, and *S. mansoni* by means of faecal egg counts (FECs; expressed in eggs per gram of stool (EPG)) for each individual and each pooled sample. Subsequently, the agreement between mean FEC based on the examination of individual samples and the FEC based on the examination of the pooled sample was evaluated by the Pearson’s correlation coefficient (R). In addition, a permutation test (10,000 iterations) was applied to test for differences in mean FEC between examination of individual and pooled samples. The level of significance was set at *p* < 0.05.

#### Assessment of time to examine stool samples

We calculated the total time to prepare and examine individual and pooled samples, using the formulae described in Table 1. The 95 % confidence intervals (95 % CI) were obtained by bootstrap analysis (10,000 iterations).

**Table 1:** Formula to calculate the time to quantify soil-transmitted helminth and *Schistosoma mansoni* eggs in stool based

	Individual samples (*n* = 360)	Pooled samples		
		Pool size of 5 (*n* = 72)	Pool size of 10 (*n* = 36)	Pool size of 20 (*n* = 18)
Pooling stool samples	_	72 x mean time to make one pool of 5	72 x mean time to make one pool of 5 + 36 x mean time to make one pool of 10	72 x mean time to make one pool of 5 + 36 x mean time to make one pool of 10 + 18 x mean time to make one pool of 20
Preparing of KK smears	+36 x mean time to prepare 10 KK thick smears	+ 7.2. x mean time to prepare 10 KK thick smears	+ 3.6 x mean time to prepare 10 KK thick smears	+ 1.8 x mean time to prepare 10 KK thick smears
Examining of KK smears	+ Total time required to examine 360 KK thick smears of 360 individual stool samples	+ Total time required to examine 72 KK thick smears of 72 pools of 5	+ Total time required to examine 36 KK thick smears of 36 pools of 10	+ Total time required to examine 18 KK thick smears of 18 pools

*KK* Kato-Katz

## Results

### Prevalence and intensity of STH and *S. mansoni* infections

Eggs of either STHs or *S. mansoni* where found in 218 out of the 360 (60.5 %) subjects screened. *T. trichiura* was the predominant species (30.6 %), followed by *S. mansoni* (25.3 %) hookworm (21.4 %) and *A. lumbricoides* (18.1 %). The arithmetic mean FEC was 2,596.3 EPG, 126.0 EPG, 47.3 EPG and 40.7 EPG for *A. lumbricoides, T. trichiura*, *S. mansoni* and hookworm, respectively. Across schools, there was large variation both in prevalence and infection intensity for each of the four helminth species (Table 2). The most pronounced variation in prevalence between schools was observed for both *A. lumbricoides* (1.7 %–43.3 %) and *S. mansoni* (6.7 %–46.7 %), where the range in prevalence was approximately 40 %. The least variation in prevalence was observed for hookworm (8.3 %–31.7 %).

**Table 2:** The prevalence and infection intensity of *Ascaris lumbricoides*, *Trichuris trichiura*, hookworm and *Schistosoma mansoni* infections in 6 primary schools in Jimma town and Kore village, Jimma zone, Southwest Ethiopia

School ID	Number	*A. lumbricoides*		*T. trichiura*		Hookworm		*S. mansoni*	
		Prevalence (%)	Mean FEC (EPG)	Prevalence (%)	Mean FEC (EPG)	Prevalence (%)	Mean FEC (EPG)	Prevalence (%)	Mean FEC (EPG)
School 1	60	26.7	4,661.6	45.0	289.6	8.3	13.2	23.3	54.8
School 2	60	20.0	3,279.2	38.3	284.8	10.0	12.0	13.3	31.2
School 3	60	43.3	7,218.8	40.0	126.0	18.3	31.2	30.0	36.0
School 4	60	1.7	25.2	31.7	26.4	31.7	42.0	31.7	59.6
School 5	60	8.3	83.2	16.7	10.8	26.7	110.0	46.7	98.0
School 6	60	8.3	310.0	13.3	18.4	31.7	35.6	6.7	4.4
*Total*	*360*	*18.1*	*2,596.3*	*30.8*	*126.0*	*21.1*	*40.7*	*25.3*	*47.3*

*FEC* faecal egg counts, *EPG* eggs per gram of stool

### Agreement in FECs between individual and pooled stool samples

**Fig. 3:**
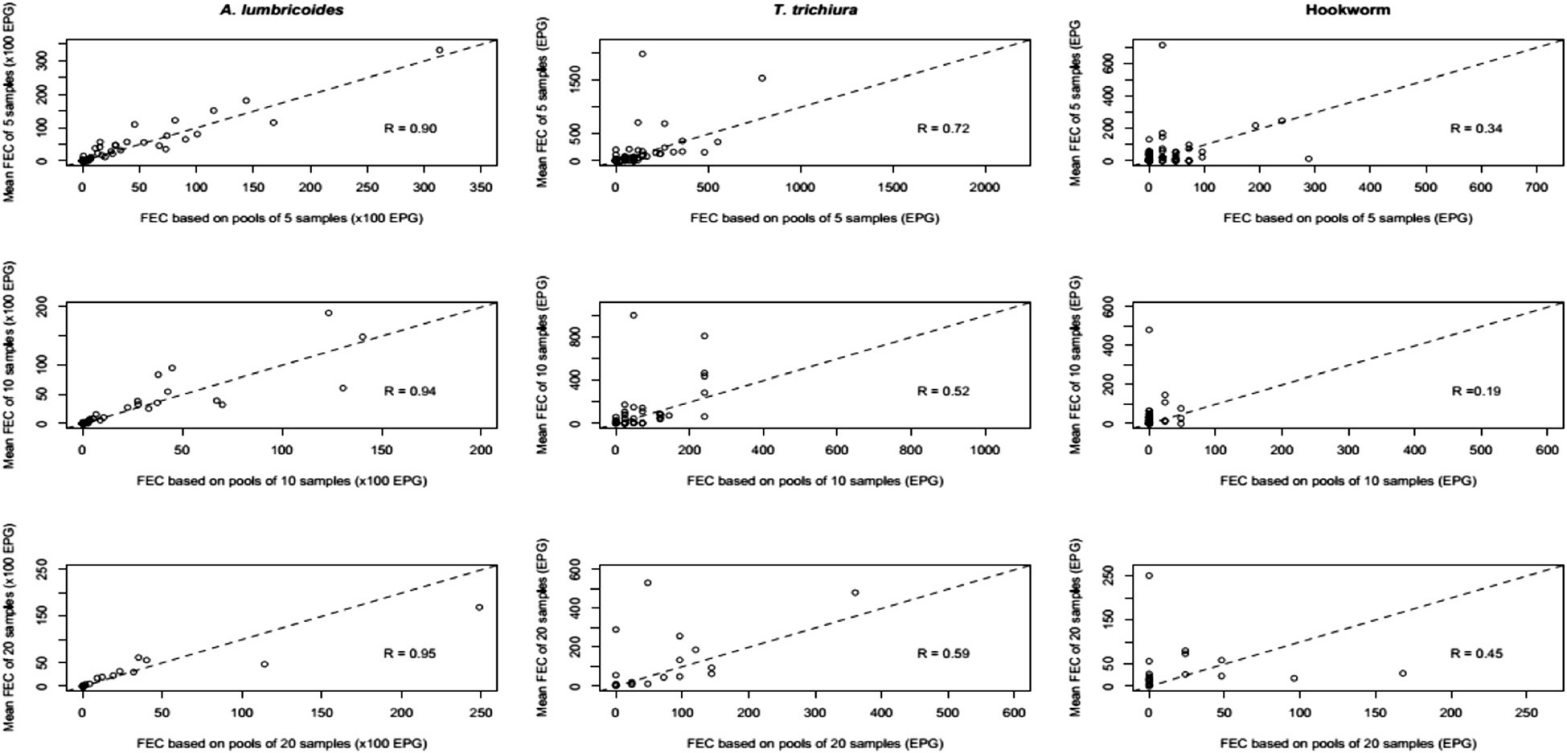
Agreement in faecal egg counts (FEC; expressed in eggs per gram of stool (EPG)) of soil-transmitted helminths between individual and pooled samples. Each of the 9 scatter plots represents the agreement in mean individual FEC and pooled FEC of stool samples. The plots in the first column represent *Ascaris lumbricoides*, second column *Trichuris trichiura*, and the third column hookworm. The plots in top, middle and bottom row represent pool sizes of 5, 10 and 20, respectively. The magnitude of correlation for each plot is based on the Pearson’s correlation coefficient (R)

**Fig. 4:**
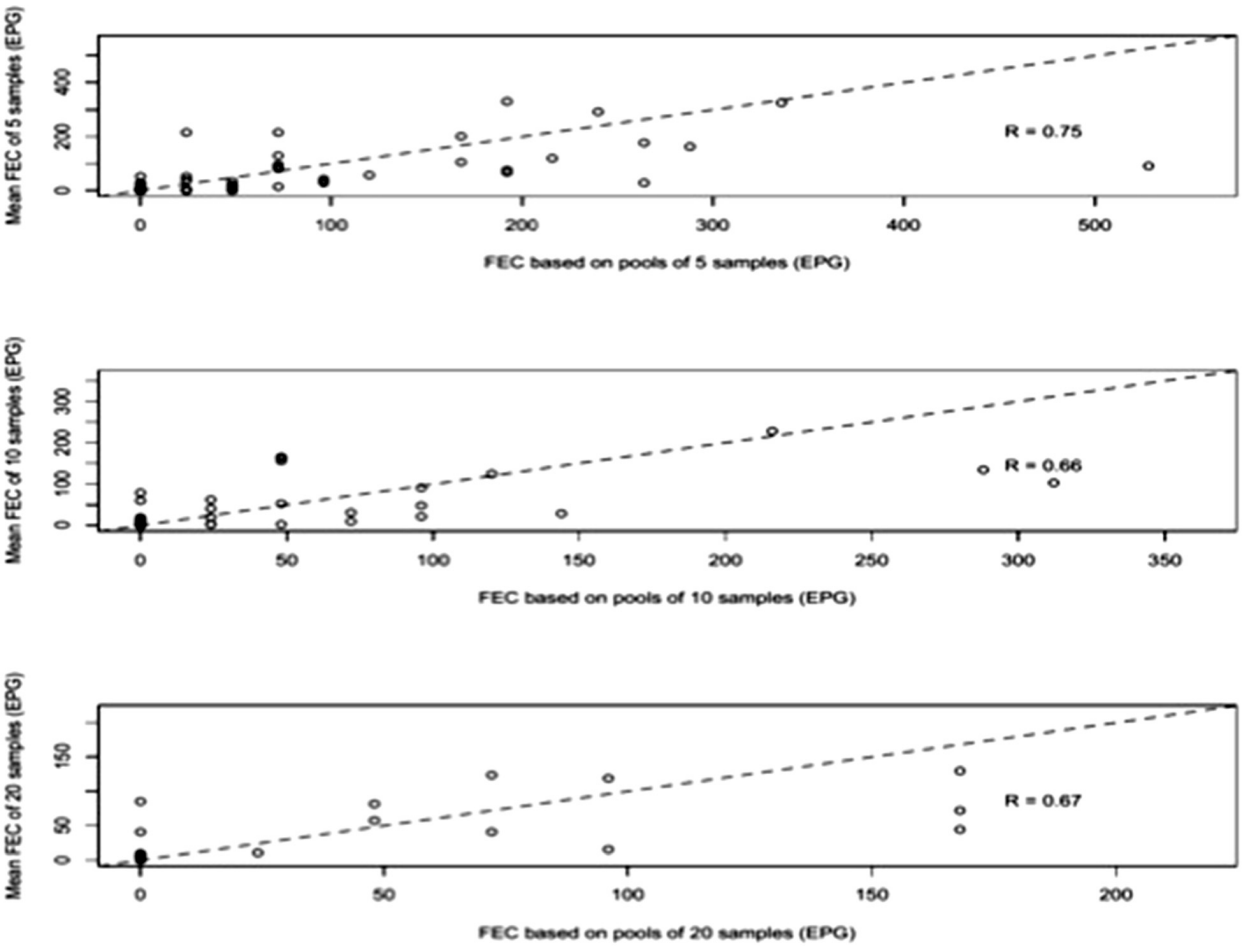
Agreement in faecal egg counts (FEC; expressed in eggs per gram of stool (EPG)) of *Schistosoma mansoni* between individual and pooled samples. Each of the 3 scatter plots represents the agreement in mean individual FEC and pooled FEC of stool samples. The plots in top, middle and bottom rows represent pool sizes of 5, 10 and 20, respectively. The magnitude of correlation for each plot is based on the Pearson’s correlation coefficient (R)

The correlation in mean FEC of individual samples and the FEC of the pooled samples for the three pool sizes is illustrated in Fig. 3 for STHs and in Fig. 4 for *S. mansoni*. Overall, there was a significant positive correlation between mean FEC of individual samples and the FEC of the corresponding pooled samples for each of the three pool sizes for *A. lumbricoides* (R_*A. lumbricoides*_ = 0.90–0.95, *p* < 0.01), *T. trichiura* (R_*T. trichiura*_ *=* 0.52–0.72, *p* < 0.01) and *S. mansoni* (R_*S. mansoni*_ = 0.66–0.75, *p* < 0.01). For hookworm, a positive correlation was found for each pool size, but only for a pool size of 5 the correlation was significantly different from zero (R_pool of 5_ = 0.34, *p* < 0.01; R_pool of 10_ = 0.19, *p* = 0.26; R_pool of 20_ = 0.45, *p* = 0.06)*.*

### Difference in mean FECs

Table 3 summarizes the mean FECs for both individual and pooled samples. Overall, there were no significant differences in FEC between individual and pooled samples for *A. lumbricoides*, *T. trichiura* and *S. mansoni.* Only for hookworm was a significant difference in FEC observed for a pool size of 10, examination of pools resulting in lower FECs (FEC_pools of 20_ = 6.7 EPG *vs.* FEC_individual_ = 40.7 EPG, *p* < 0.01).

**Table 3:** Mean faecal egg counts for *Ascaris lumbricoides*, *Trichuris trichiura*, hookworm and *Schistosoma mansoni* infections based on the examination of both individual and pooled samples (pool size of 5, 10 and 20)

Pool size	Sample size	*A. lumbricoides*		*T. trichiura*		Hookworm		*S. mansoni*	
		Mean FEC (EPG) (95 % CI)	p-value	Mean FEC (EPG) (95 % CI)	p-value	Mean FEC (EPG) (95 % CI)	p-value	Mean FEC (EPG) (95 % CI)	p-value
1	360	2,596.3 (1,688.4; 3,588.5)	_	126.0 (66.9; 206.1)	_	40.7 (23.9; 64.7)		47.3 (31.2; 66.3)	_
5	72	2,281.7 (1,271.6; 3,521.0)	0.11	95.3 (65.0; 131.0)	0.39	26.3 (15.7; 39.7)	0.23	61.7 (40.7; 86.3)	0.12
10	36	2,367.3 (1,246.0; 3,688.7)	0.54	73.3 (49.3; 100.0)	0.11	6.7 (2.7; 12.0)	<0.01	52.0 (29.3; 79.3)	0.68
20	18	3,008.0 (853.3; 6,161.5)	0.50	70.7 (36.0; 116.0)	0.11	24.0 (6.7; 46.7)	0.40	53.3 (26.7; 82.7)	0.64

*FEC* faecal egg counts, *EPG* eggs per gram of stool

### Time required to prepare and screen individual and pooled stool samples

The total time required for the examination of individual and pooled stool samples is summarized in Table 4. Overall, the total time to examine 360 individual stool samples equalled 65 h 5 min. The time (proportion of the time required to screen individual samples) required to screen pools of 5, 10 and 20 was 19 h 12 min (29.5 %), 14 h 39 min (22.5 %) and 12 h 42 min (19.5 %), respectively.

**Table 4:** Total time to quantify soil-transmitted helminth and *Schistosoma mansoni* eggs in stool from 360 school children applying Kato-Katz technique on both individual and pooled stool samples (pool size of 5, 10 and 20)

	Individual samples (*n* = 360)	Pooled samples		
		Pool size of 5 (*n* = 72)	Pool size of 10 (*n* = 36)	Pool size of 20 (*n* = 18)
Total time to pool stool samples (95 % CI)	_	6 h 35 min (6 h 22 min; 6 h 48 min)	8 h 29 min (8 h 15 min; 8 h 45 min)	9 h 20 min (9 h 4 min; 9 h 36 min)
Total time to prepare KK thick smears (in batches of 10) (95 % CI)	15 h 29 min (14 h 48 min; 16 h 10 min)	3 h 6 min (2 h 58 min; 3 h 14 min)	1 h 32 min (1 h 29 min; 1 h 37 min)	46 min (45 min; 49 min)
Total time to examine KK thick smears (95 % CI)	49 h 36 min (46 h 50 min; 52 h 26 min)	9 h 30 min (8 h 52 min; 10 h 15 min)	4 h 37 min (4 h 17 min; 4 h 59 min)	2 h 36 min (2 h 15 min; 3 h 4 min)
Total time (95 % CI)	65 h 5 min (62 h 13 min; 67 h 58 min)	19 h 12 min (18 h 29 min; 19 h 57 min)	14 h 39 min (14 h 14 min; 15 h 7 min)	12 h 42 min (12 h 15 min; 13 h 14 min)

*KK* Kato-Katz, *95 CI* 95 % confidence intervals

## Discussion

A scale-up of MDA programmes to control the morbidity caused by STH and *S. mansoni* infections is underway in various parts of Africa, Asia and South-America. Periodically assessment of these infections, however, remains crucial to inform programme managers, healthcare decision makers and donors of the drugs to assess whether objectives are being met, and if necessary, to adjust the strategy implemented. Our study highlights that pooling of stool samples could translate in important cost-savings in large-scaled epidemiological surveys required to monitor progress of these MDA programmes. First, our results confirm that pooling stool samples provided intensity estimates for both STH and *S. mansoni* infections comparable to those obtained after examination of individual stool samples when using the Kato-Katz technique. These findings of comparable infection intensity estimates are in line with previous studies assessing pooling strategies to evaluate helminth infections in both animals [12–15] and humans [8]. Second, it shows that time for processing and examination of samples using Kato-Katz can significantly be reduced, suggesting that the same funds would support examination of 3 to 5 times more subjects when individual stool samples are pooled into pools of 5 or 20, respectively. Note that these estimates do not include the additional time required for data entry, and hence are likely to be underestimated. Screening a larger number of subjects without compromising on the accuracy of infection intensity estimates would allow more schools across different geographic locations to be included, and hence improve the precision of helminth disease mapping and prediction. This is particularly important given the large endemic areas and the focal distribution of these diseases [16–18].

To our knowledge this is the first study that provides time estimates for making pools of individual stool samples. Although further research will be required to verify the inter laboratory differences in making these pools, they will become very helpful to verify the cost-effectiveness of pooling stool in different scenarios of endemicity. Recently, Levecke *et al* [19] described a general mathematical framework for egg counts in stool from which one can derive the required sample size for assessing the population mean FEC with a predefined level of precision for any scenario of endemicity (mean population FEC and aggregation of FEC between individuals) and diagnostic strategy (amount of stool examined ~ sensitivity of the diagnostic technique) and examination of individual/pooled samples) (https://paradesign.shinyapps.io/paradesign/). Combining the time estimates both for making pools (the present study) and processing stool samples for different diagnostic techniques available in literature [20, 21], programme managers and health-care decision makers can now compare different diagnostic strategies and their corresponding technical and financial resources required, and hence optimize the use of funds allocated for monitoring MDA programmes to control STH.

A common criticism on pooling samples is that it does not allow estimation of the prevalence. Indeed, the proportion of pools in which eggs are detected does not provide an unbiased estimate of the true underlying prevalence [22], yet prevalence remains a key measurement for initiation and scaling down MDA programmes [2]. To overcome this, various researchers have developed statistical methods to estimate the true underlying prevalence based on the examination of pooled samples [19, 22, 23]. In addition, there is on-going debate whether prevalence is an appropriate measure to monitor the long-term impact of helminthiasis control programmes, as opposed to infection intensity. Anderson and colleagues [24], highlighted that a drop in FEC may not always be reflected in a drop in prevalence, and hence underestimating the impact of control interventions.

Although we have broadened the basis for implementing a pooling strategy in MDA programmes, there are a few aspects that require further attention. First, we did find a significant difference in mean FEC for hookworms, suggesting that pooling may not always provide accurate estimates of infection intensity. A significant difference in mean FEC for hookworms between an individual and pooled examination strategy was not observed in a previous study in the same geographical area, but using McMaster egg counting method instead [8]. A possible explanation for this discrepancy is the impact of the malachite-green/glycerine solution on hookworm eggs in slides of the pooled samples. Kato-Katz smears should be read within 30 to 60 min after preparation to avoid that hookworm eggs will collapse or become distorted, and hence resulting in underestimation of FEC or even resulting in false negative test results [9–11, 25]. Second, there is decrease in mean FEC as a function of increasing pool size that is most pronounced for *T. trichiura* and hookworm infections. This trend might be due to the lack of sensitivity of the Kato-Katz when FEC are low [26, 27]; the more samples are pooled the lower the expected FEC in the pool is, but it remains unclear why this would not apply for *S. mansoni* for which overall egg excretion in this setting is low too. Third, current models to estimate the true underlying prevalence based on pooled samples needs to be thoroughly validated. Finally, to further reduce the work in the laboratory or in the field, it would be important to also verify the validity of pooling samples when pools are made based on a fixed volume of stool rather than a fixed mass.

## Conclusions

Our results indicate that pooling of stool sample holds promise as a rapid assessment of infection intensity for both STHs and *S. mansoni* by Kato-Katz technique. In this setting, time in the laboratory was reduced with 70 % when pools of 5 instead of individual stool samples are screened, and hence pooling of stool could translate into important cost-savings in large-scaled epidemiological surveys to monitor progress of MDA programmes.

## Abbreviations

MDA: Mass drug administration
WHO: World Health Organization
STH: Soil-transmitted helminths
FEC: Faecal egg counts
EPG: Eggs per gram of stool
R: Pearson’s correlation coefficient
95 % CI: 95 % confidence intervals
KK: Kato-Katz
